# RNA-mediated immunotherapy regulating tumor immune microenvironment: next wave of cancer therapeutics

**DOI:** 10.1186/s12943-022-01528-6

**Published:** 2022-02-21

**Authors:** Poonam R. Pandey, Ken H. Young, Dhiraj Kumar, Neeraj Jain

**Affiliations:** 1 Department of Thoracic & Cardio Surgery-Rsch, The University of Texas MD Anderson Cancer Center, Houston, TX 77054 USA; 2 Department of Genetics and Development, Herbert Irving Comprehensive Cancer Center, Columbia University, New York, NY 10032 USA; 3 Division of Hematopathology, Duke University Medical Center, Duke Cancer Institute, NC 27710 Durham, USA; 4 Division of Cancer Biology, CSIR-Central Drug Research Institute, Lucknow, Uttar Pradesh 226031 India; 5 Academy of Scientific and Innovative Research, Ghaziabad, Uttar Pradesh 201002 India

**Keywords:** Cancer, RNA, Nanoparticle, Antibody, Dendritic cells, T cells, Cytokine, Tumor immune microenvironment, RNA therapy, Immunotherapy

## Abstract

Accumulating research suggests that the tumor immune microenvironment (TIME) plays an essential role in regulation of tumor growth and metastasis. The cellular and molecular nature of the TIME influences cancer progression and metastasis by altering the ratio of immune- suppressive versus cytotoxic responses in the vicinity of the tumor. Targeting or activating the TIME components show a promising therapeutic avenue to combat cancer. The success of immunotherapy is both astounding and unsatisfactory in the clinic. Advancements in RNA-based technology have improved understanding of the complexity and diversity of the TIME and its effects on therapy. TIME-related RNA or RNA regulators could be promising targets for anticancer immunotherapy. In this review, we discuss the available RNA-based cancer immunotherapies targeting the TIME. More importantly, we summarize the potential of various RNA-based therapeutics clinically available for cancer treatment. RNA-dependent targeting of the TIME, as monotherapy or combined with other evolving therapeutics, might be beneficial for cancer patients’ treatment in the near future.

## Background

**Fig. 1:**
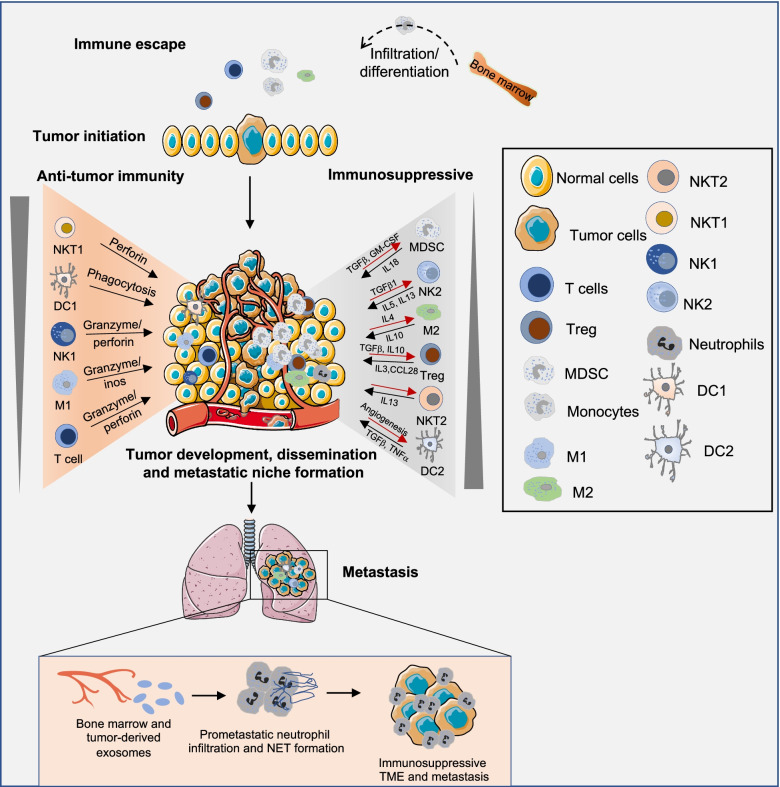
Crosstalk in the TIME during tumor development. The processes of tumor initiation, expansion, and metastasis are governed by the TIME, where immunosuppressive and antitumor immune crosstalk play an important role. During the tumor initiation stage, cancer cells escape from immune surveillance. While tumor expansion, contact-dependent or independent crosstalk between tumor and TIME affects the production of various cytokines that help in the polarization of antitumor immune response in the immunosuppressive TIME. During metastasis, tumor-derived exosomes help in the requirement and arrangement of immunosuppressive immune cells for favorable premetastatic niche formation and growth of metastases. TGF-β, transforming growth factor-β; GM-CSF, granulocyte–macrophage colony-stimulating factor; CCL28, C–C chemokine ligand type 28; IL, interleukin; TNF-α, tumor necrosis factor α

Targeting tumor cells or the tumor microenvironment (TME) are the two major fundamental principles for antitumor therapies. Therefore, understanding the TME and its immune cell components are equally important as cancer cell characteristics for tumor eradication. The TME is a dynamic, heterogeneous, and complex network consisting of tumor cells and surrounding accessories including blood vessels, immune cells, fibroblasts, adipocytes, and signaling molecules in addition to extracellular matrix components [1]. These tumors and accessories represent the hallmark characteristics that support tumor progression and lead to metastasis. The oncogenic communication with tumor cells and through the crosstalk of autocrine and paracrine components in almost all tumor types are responsible for this phenomenon. Different tumor types can also design their specific microenvironment by encouraging tumor angiogenesis and stimulating peripheral immune tolerance. Tumor-infiltrating immune cells are an important component of the TIME and are a significant predictor of cancer patients’ survival. Depending on the tumors type and stage, the infiltrating immune cells that define the fate of tumor growth can be protumor, such as neutrophils and tumor-associated macrophages (TAMs), and antitumor, such as cytotoxic CD8 + T cells and natural killer (NK) cells [2–5]. The ratio of pro- to antitumor immune populations in the TIME plays a critical role in the regulation of tumor progression and metastasis. The crosstalk between pro-tumorigenic immune cells, stromal cells, and cytokines helps to establish the pre-metastatic niche for disseminated circulatory tumor cells and facilitates metastasis. The complex interplay between cancer cells and the TIME influences the outcome of immunotherapy and other anticancer therapy (Fig. 1).

The stromal component interacts with tumor cells in complex crosstalk to support tumor growth. The functional complexity of the TIME is defined by several molecular entities such as growth factors, cytokines, and proteases originating in tumor cells and the stromal compartment during cancer progression [6]. Interestingly, several studies have also shown that intracellular or extracellular RNA molecules, including non-coding RNAs, expressed in either immune or tumor cells can regulate tumor immunity [7–9]. Studies have shown that the tumor exosomal microRNAs (miRNAs or miRs) such as miR-934 and miR-183 help in macrophage M2 polarization or promote the secretion of proinflammatory cytokines that promote tumor growth and metastasis [10, 11]. Interestingly, recent studies show that double-stranded RNA derived from tumor cells promotes the chemotactic signaling pathway in a stromal component that drives intravasation and metastasis [12]. The modification of RNA also plays a critical role in immune regulation during tumor progression. Studies from Shen et al. using The Cancer Genome Atlas database revealed that N6-methyladenosine (m6A)-mediated RNA methylation correlates with several TIME phenotypes such as immune infiltration, rejection, and deficiency in hepatocellular carcinoma [9]. These studies indicate a promising role of RNA in the regulation of TIME phenotypes that support tumor progression and metastasis. The knowledge of this complex interplay between tumor and immune cells could provide RNA as advance in therapeutic target. The new combination treatments of immunotherapy and RNA-based targeted therapies will help to overcome tumor immune evasion mechanisms and optimize the clinical benefit of current immunotherapies. In this review, we have emphasized RNA-based therapies that have clinical potential to target the spatial architecture of the TIME, recommendations to overcome current setbacks, and future therapeutic developments in the field of cancer biology (Fig. 1).

## TIME and current immunotherapies

The immune cells within the TME are a critical component that can reprogram and are thought to control the growth, evasion, metastasis, and evolution of cancer cells, resulting in clinically unresponsive tumor development [6, 13]. The TIME is broadly populated with immune cells including myeloid cells (myeloid-derived suppressor cells [MDSCs], TAMs, and neutrophils), lymphocytes (CD4 + T helper cells, CD8 + cytotoxic T cells [CTLs], regulatory T cells [Tregs], and NK cells), antigen-presenting cells (including B cells and dendritic cells [DCs]), cell-surface molecules (cytokines receptors), immune checkpoints (ICPs), and non-cellular components including soluble immune factors such as cytokines, chemokines, and growth factors. The composition of immune cells in the TIME varies depending upon the tumor type and differs among patients of the same tumor type, creating new challenges in the field of cancer biology. Therefore, deeper analysis of TIME complexity will reveal novel biomarkers that will be fruitful in current therapy modulations. Among immune cells in TIME, antigen-presenting cells, NKs, and CTLs act as tumor suppressors, while TAMs, Tregs, and MDSCs promote immunosuppressive roles and help in tumor progression. Due to genetic alterations in tumors and immune cells, CTLs and NK cells have limited efficacy in the TME [14]. Additionally, the presence of immunosuppressive cells and/or accumulation of oncogenic cytokines may suppress the functions of effector immune populations [14]. Advances in technologies such as immunoscore, multiplexed flow cytometry, histological slide scanning, co-detection by indexing, multiplexed ion beam imaging, and high-resolution single-cell RNA sequencing have revolutionized the understanding of immunology in TIME. These technologies have enabled not only deciphering the TIME’s molecular features and composition but also elaboration of its diversity, complexity, and spatial architecture, revealing classes and subclasses of the TIME and its influence on response to therapy [7, 13].

The current clinical scenario of cancer patients presents two major obstacles: (i) immune escape and (ii) acquired therapy resistance, which are also associated with the immunosuppressive microenvironment. Therefore, reprogramming the TIME and reversing immunosuppressive strategies will likely benefit current cancer treatment modalities. Different strategies have been adopted in cancer immunotherapy, which has progressed in last decade. The first generation of cancer immunotherapy involves but is not limited to the use of immunostimulatory cytokines such as IFN-α and IL-2, which induce the host antitumor response [15–17]. However, due to low response rates and associated toxicities at high doses, in clinical practice these cytokines have been largely displaced in favor of ICP inhibitors or targeted therapy [16]. The second generation of cancer immunotherapy uses ICP inhibitors in combination with immunological cell death inducers or chimeric antigen receptor (CAR) T cells, which are designed to inhibit specific immunosuppressive molecules/cells, stimulate specific cellular processes, or target-specific tumor cells, resulting in effective antitumor response [18, 19]. The third generation of cancer immunotherapy consists of combination strategies targeting ICP and the TIME, which is expected to suppress the multiple aspects of negative immune regulation, increasing treatment effectiveness and providing a safe antitumor response [1]. To date, ICP-targeting drugs such as ipilimumab, which targets cytotoxic T-lymphocyte-associated protein 4 (CTLA-4); nivolumab, which targets programmed cell death protein 1 (PD-1); and atezolizumab, which targets PD-1 ligand 1 (PD-L1) have been shown to be effective cancer immunotherapies, especially in solid malignancies [18, 20–23]. Similarly, CD19-directed CAR T-cell therapy achieved 70–90% rates of complete remission in B-cell lymphoma patients [24]. Despite of these research breakthroughs, subsequent studies have demonstrated that in clinical settings, complete responses are limited by acquired resistance, loss of drug target, and primary refractoriness to these agents [24]. For example, downregulation of CD19 expression is the major cause of treatment failure in CD19-specific CAR T-cell therapy [25]. Furthermore, clinical use of these drugs potentially can elicit high-grade immune-related adverse effects [26]. ICP blockade (ICB) antibodies and cell-based therapeutics such as CAR T cells in tumor immunotherapy are in their infancy, and it is desirable to discover new strategies for improving their safety and efficacy, along with alternative strategies.

## RNA molecules: alternative immunotherapy for cancer

**Fig. 2:**
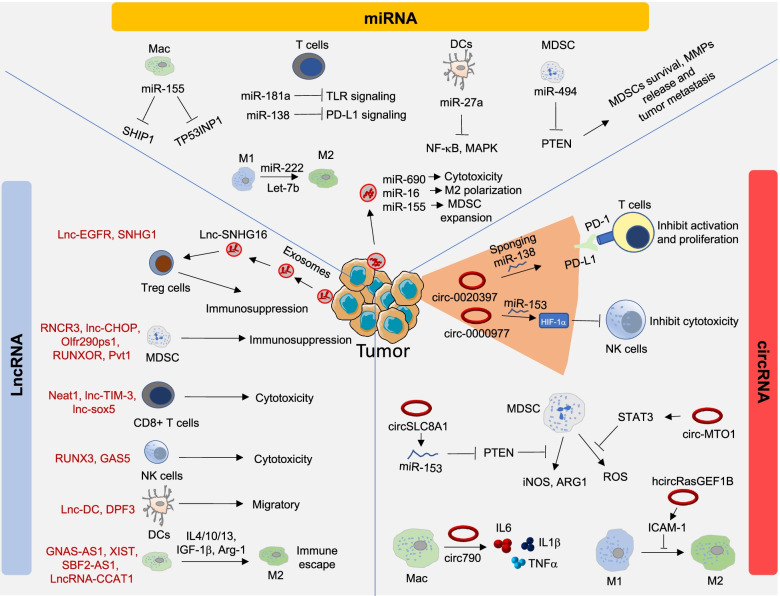
Illustration of non-coding RNAs in the modulation of the TIME during tumor growth and progression. miRNAs play an important role in regulating TIME functions that control several oncogenic signaling, secretome, and ICP molecules. CircRNAs have been shown to play roles during tumor progression such as proliferation, growth, invasion, and metastasis. The tumor exosomes that also contain circRNAs help in the modulation of TIME function and promote the immunosuppressive TME during tumor growth. circ-0020397 and circ-0000977, inhibit T and NK cell activation in the TIME. In addition, circRNAs in the immune cells contribute to the development of the immunosuppressive TME. During tumor growth and metastasis, lncRNAs play critical role in immune escape in the TIME. In the TME, lncRNAs control various immune and cancer cell crosstalk signals that promote the activation of immunosuppressive cells such as MDSCs and TAMs. Moreover, lncRNAs such as Neat1 and RUNX3 block the cytotoxic activity of CD8 + T cells and NK cells, respectively

Recently, Yeo’s group has investigated the function of several RNA binding proteins using CRISPR-Cas9 screening in cancer and identified 57 RNA binding protein candidates with critical roles in promoting MYC-driven oncogenic pathways [27]. This study highlights the therapeutic applicability of RNA binding proteins by discovering the essential role of YTHDF2 protein in the global transcription regulation of MYC-driven breast cancers [27]. Besides targeting protein-coding entities for cancer treatment, non-coding entities such as miRNAs, long non-coding RNAs (lncRNAs), and circular RNAs (circRNAs) exhibit important functions in the TME modulation. These non-coding entities function at chromatin level or post-transcriptional regulation of gene expression, which in turn modulates oncogenic transformation and other pathophysiological processes [28]. Hence, new avenues of RNA therapeutics are gaining attention among researchers. Compared to conventional approaches such as small molecular drugs/inhibitors or peptide-/protein-specific therapeutic antibodies, RNA therapeutics play a regulatory role in cancer treatment by controlling the expression of target proteins at varying degrees. Moreover, RNA therapeutics are easier to design than molecular or protein-based drugs. Hence, RNA-based approaches are an attractive option in molecular medicine research and provide a rationale for their clinical application in cancer treatment. In 2016, The US Food and Drug Administration (FDA) approved the antisense oligonucleotide (ASO) drug nusinersen, a splicing modulator, to treat spinal muscular atrophy affecting children [29]. This success story was followed by another, when the first RNA interference (RNAi) drug patisiran (target abnormal form of the protein transthyretin) received FDA approval in August 2018 for the treatment of polyneuropathy [30]. Interestingly, RNA molecules (small interfering RNA [siRNA], microRNA [miRNA], and messenger RNA [mRNA]) have shown immunomodulatory effects, indicating potential for cancer immunotherapy [31, 32]. Hence, RNA-based therapy is highly desirable and has become a trending subject matter/field of research in immunotherapy (Fig. 2). These therapies stimulate both innate and adaptive immunity by silencing or upregulating immune-relevant genes (e.g., silencing ICP genes), regulating cytokine expression, and functioning as tumor antigen vaccines [31–33].

The use of RNA-based therapeutics is expanding dramatically, and some of them have been tested in clinical trials, revealing their possibility for cancer treatment. However, their clinical application is limited by their lack of stability, toxicity, and various physiological factors that inhibit their intracellular delivery [33–35]. An innovation that may solve these barriers to delivery of RNA therapeutics is nanoparticle (NP)-based platforms such as liposomes, polymeric NPs, and inorganic NPs [36–39]. These advances have paved the way for RNA-based therapeutics in immunotherapy for cancer patients (Fig. 2).

## Targeting TIME using RNA-based platforms

### Current knowledge toward mRNA-based cancer immunotherapy

mRNA-based therapy has emerged as a better option than conventional [40–42] and recombinant protein-based [41] gene therapy because it uses endogenous cell machinery for full-length natural and higher-magnitude protein production. In addition, advancements in structural designing of mRNA molecules and improved pharmaceutical formulations for in vivo stability and selective target have significantly enhanced the therapeutic activities of mRNA. In general, mRNA therapies consist of specific mRNA sequences delivered into the patient’s body, which uses cellular machinery to synthesize specific folded proteins involved in the development of disease. Currently, mRNA has been broadly used as a powerful tool for treating various human diseases, especially malignant tumors [41]. However, in vitro transcribed, or synthetic mRNA molecules are unstable and easily degraded by ribonucleases. The positive results from various preclinical tumor models suggested that in vitro transcribed mRNAs can be expanded to generate passive cellular immunotherapy [43, 44]. By targeting multiple tumor-specific neoantigens, mRNA-based treatment strategies could elicit antitumor immune responses from the innate and adaptive immune systems with alleviated HLA restrictions [45, 46]. An interesting study led by Lin et al. revealed that PTEN mRNA delivered by NPs efficiently reactivates the tumor suppressor PTEN in Pten-mutated melanoma cells and Pten-deficient prostate cancer cells. PTEN reactivation leads to autophagy, which triggers the release of damage-associated molecular patterns. These molecules reverse the immunosuppressive TME and induce cell death in cancer by enhancing CD8 + T cell infiltration and reduction of Tregs and MDSCs [47]. Therefore, advanced protocols for the transport of tumor suppressors provide an opportunity to improve antitumor immune responses in the TIME [47]. The major challenge of using mRNA-based treatments are their instability and easy degradability by ribonucleases. In the past decade, multiple innovations have improved mRNA stability which had made it a more feasible candidate for vaccine development. mRNA-based therapeutics for immuno-oncology, protein replacement therapies, and RNA vaccine development have significantly improved, and as a result, more than 20 mRNA-based cancer immunotherapies have entered clinical trials with some promising treatment results [33, 48, 49].

### Cancer RNA vaccines and clinical trials

As mentioned before, RNA-based vaccines are promising options for combination treatment with conventional vaccines. The first successful human phase 1 clinical trial (NCT02410733) of an RNA vaccine for melanoma, FixVac (BNT111), encouraged researchers to develop more RNA-based cancer vaccines [33, 41, 45, 50]. FixVac is a nanoparticulate liposomal RNA that encodes four tumor-associated antigens (NY-ESO-1, MAGEA3, tyrosinase, and TPTE) that are commonly expressed in melanomas and are highly immunogenic. FixVac targets DCs in vivo and induces antigen presentation, which further leads to induction of an effector T cells response against melanoma-associated antigens and suppresses tumor growth. Importantly, though the FixVac is active as a single agent, it showed a synergistic effect with ICP inhibitors [51]. Besides using tumor antigens as the target for mRNA-based therapy, the addition of an mRNA encoding immunostimulants as therapy could elevate the activation of antitumor immunity. For instance, an ex vivo autologous monocyte-derived DC vaccine was developed by Argos (Rocapuldencel‑T). In this vaccine, DCs are transfected in bulk with tumor-antigen mRNA from the individual patients and are activated by co‑transfecting with CD40L mRNA that are primarily present in activated T cells [52]. Furthermore, Rocapuldencel‑T has shown a promising effect in phase 2 trial (NCT00678119) in stage IV renal cell carcinoma (RCC) when combined with sunitinib mRNA therapy [52]. Though, the vaccine induced immune response but did not improve the overall survival of RCC patients in the ADAPT phase 3 trial (NCT01582672) [53]. Various RNA-based vaccines have been extensively used to modulate the TIME by engineering tumor-associated DCs or suppressor cells or modifying cytokines that eventually activate cancer-specific T cells and lead to cancer cell death. A number of completed and ongoing clinical trials have extensively explored a group of in vitro transcribed mRNA-based immunotherapies encoding either immunostimulants (e.g., IL-12, CD40L, CD70), or tumor-associated antigens, or neoantigens [54]. A phase 1b clinical trial (NCT01915524) in patients with stage IV non-small cell lung cancer (NSCLC) indicated the benefits of sequence-optimized mRNA vaccine BI1361849 (CV9202) combined with radiotherapy [48]. BI1361849 encodes six NSCLC-associated antigens, (NY-ESO-1, MAGE-C1, MAGE-C2, survivin, 5T4, and mucin 1) and was shown to upregulate targeted immune responses. In a similar line, a phase 1/2 clinical study (NCT03164772) is exploring the safety and efficacy of BI1361849 combined with ICP inhibitors durvalumab (anti-PD-L1) and tremelimumab (anti-CTLA-4) in NSCLC patients [55].

Many advanced mRNA-based protocols are being developed for novel cancer immunotherapies. A recent study demonstrated the antitumor immunity and tumor eradication ability of mRNA-based therapy in multiple preclinical tumor models. Saline-formulated mRNA for four cytokines gene (IL-2, IL-15, IFN-α, and GM-CSF) when administered at the tumor site led to systemic antigen-specific T cell expansion, granzyme B + T cell infiltration, and immunological memory development. Moreover, profound tumor regression was identified when this mRNA-based cytokine therapy was combined with immunomodulatory agents [56].

### Molecular implications of alternative splicing events and cancer immunotherapy

Another advanced approach called alternative mRNA splicing is a new category of pre-mRNA transcript technology having clinical applicability. Alternative processing or splicing of mRNA alters the total RNA pools of the transcript, creating proteomic diversity (i.e., neoantigens) in cancers; this diversity offers promising immunotherapeutic targets. Polyadenylation-the addition of poly-A tail at 3’end of mRNA is a complex process, and some mRNA transcripts are often alternatively polyadenylated [57]. The alternative polyadenylation (APA) at 3ʹ untranslated region (UTR) regulates the stability, localization, and translation of a transcript [58]. However, the APA events that occur in upstream intronic regions called intronic polyadenylation (IPA) of the last exon generate either non-coding transcripts and truncated coding regions, both of which have been linked with tumor progression [59–62]. Concerning cancer immunotherapy, the identification of IPA events is critically important in the discovery of new tumor-specific peptides because tumors bear more alternative splicing events than healthy tissues [63]. It has been shown that IPA events commonly occur in genes that affect cancer progression, making them immunotherapeutic targets [64, 65]. The new peptides/neoantigens generated by IPA events can be presented on MHC molecules and recognized by the immune system; therefore, tumor-specific IPA peptides that interact with MHC molecules need to be explored. The 3ʹ seq, which is used to identify and quantify polyadenylation site usage, could help us to identify global changes in 3’ UTR landscape during malignant transformation and define immunotherapeutic target space [66]. This might also serve as a predictive biomarker in response to ICP blockade. In vitro and in vivo studies have shown that small molecule inhibitors can act at different stages of the splicing process. For example, targeting splicing factor 3b (SF3b) using FR901464, a natural product that inhibits pre-mRNA splicing, results in antitumor response [67, 68]. This finding suggests that the correlation of altering APA and immunotherapy is a promising and is new area of research. Targeting genes that undergo altered APA in combination with immunotherapy in cancer or the TIME may be a novel approach to combat tumor growth. Besides APA, RNA methylation at the N6 position of adenosine (m6A) also regulates protein expression through splicing, translation, degradation, and export and thereby modulates the TIME [69, 70]. Notably, alternative splicing in immune cells that generate altered immunostimulants could also improve antitumor functions of immune cells [71].

### ASO-mediated targeting of TIME

ASOs are single-stranded, chemically synthesized nucleic acids, ~ 18–30 nucleotides long [72, 73]. These can act as small molecule drugs that target RNAs and regulate gene expression by complementary base pairing and interfering with various steps such as splicing, transcription, export, or translation through different mechanisms [74–76]. Depending upon their mode of action, ASOs are divided into two main categories, the first by promoting RNase H1 cleavage and Argonaute 2 degradation and the second by steric hindrance-mediated regulation, referred to as steric block [73, 74]. The RNase H1-based ASOs are used for targeting nuclear transcripts such as pre-mRNAs and lncRNAs, which are non-degradable by siRNA. Steric block ASOs act by modulating the different stages of RNA processing and the interactome of the target RNA [77, 78].

More recently, RNA-based therapeutic strategies have been revolutionized by combinatorial approaches using ASOs to regulate protein expression in different diseases models, including cancer [72, 75]. Further studies have revealed the application of ASOs to target microRNAs (and other noncoding RNA regulators), as well as to regulate alternative splicing of transcripts, are an efficient approach to regulate protein expression [79]. ASOs can be used to induce isoform switching to produce therapeutic/beneficial proteins and/or to inhibit the expression of harmful proteins associated with cancer/disease progression. Based on these mechanisms, the FDA has approved the splice switching ASOs golodirsen, nusinersen, and eteplirsen to control disease progression [76]. The locked nucleic acid (LNA)-modified ASOs have already been tested to decrease metadherin (MTDH) expression which promotes colorectal, lung, and breast cancer growth and metastasis. An LNA antisense ASO was shown to target and effectively suppress MTDH expression, thereby helping avoid cytotoxic T cell exhaustion and inhibiting cancer growth under in vitro and in vivo conditions [80]. The ASO-based gene expression regulation approach has provided new tools to target oncogenic genes for which no therapeutic molecules had been available. For example, glycine decarboxylase (GLDC) gene is often upregulated in lung, brain, prostate, and other cancers and provides a growth advantage to cancer cells by regulating glycine catabolism during nucleotide synthesis. Hence, splice-modulating steric-block ASOs were specifically developed to target GLDC, which promoted exon-skipping to disrupt the open reading frame of GLDC transcripts and subjected it to nonsense-mediated degradation. These GLDC steric block ASOs reduced proliferation and colonization in lung cancer cells and reduced the xenografts tumor growth in mice [81]. Ge et al. recently discovered phosphorothioate ASOs that inhibited miR-21 expression, which in turn downregulated the proliferation of NSCLC cells by inducing apoptosis through activation of the caspase-8 pathway [82]. Other findings indicate that tumors with mutant KRAS exhibit a more efficient response to ICB therapy. However, oncogenic KRAS promotes immune escape and immune therapy resistance through attracting immune-suppressive cells or suppressing cytotoxic cells [83]. Therefore, more advanced, or alternative therapies are needed to improve ICB immunotherapy to improve clinical outcomes. Another emerging RNA-based therapy, AZD4785, a high-affinity KRAS mRNA-targeting ASO that effectively decreases mutant KRAS has gain lot of interest recently. It downregulates the effector pathways and selectively decreases the proliferation of cells harboring mutant KRAS [84]. In addition, systemic injection of AZD4785 in NSCLC mice and patient-derived xenografts harboring mutant KRAS inhibited its expression and showed strong antitumor activity. Due to the limitation of subcutaneous and patient-derived xenograft models of lung cancer, study did not explore antitumor immunity in an intact immune microenvironment [84].

Studies suggest that activation of PI3K/Akt pathway in tumor cells affects several cytokines and inflammatory factors production in TIME. These changes lead to enhance the immunosuppressive activity of MDSCs and increase the expression of metalloproteinases (MMPs) that promote the immune escape and metastasis of tumor cells [85]. Moreover, it has been well established that high expression of Bcl-2 and Akt help in human cancer progression. Under the preclinical model, Cheng et al. have demonstrated that targeting Bcl2 and Akt-1 with ASOs G3139 (oblimersen) and RX-0201 respectively showed greater antitumor activity, longer survival time in the lung xenograft model [86]. In a similar line, advanced G3139 and RX-0201 ASOs, are being developed for their efficacy and safety in clinical trials to modulate TIME [86]. Signal transducer and activator of transcription 3 (STAT3) have been associated with the aggressive phenotype of cancer. STAT3 is a ubiquitously expressed transcription factor and master regulator of immune suppression. The polarization of protumor macrophage and MDSCs are regulated by STAT3 transcript factor [87]. While multiple therapies are being used to target STAT3 signaling, there has been limited selectivity to target STAT3 specifically. Alternatively, STAT3 can also be targeted by ASO, AZD9150 that decreased the expression of STAT3 and conferred promising antitumor effects in several preclinical cancer models of lymphoma and lung cancer [87]. Using the isograft model, Proja et al. have shown that a combination of STAT3 ASO and anti-PD-L1, remodel the immunosuppressive microenvironment that led to enhanced T- cell abundance and exhibit antitumor response [87]. Interestingly, data from phase 1 clinical trial (NCT01563302), AZD9150 was well endured and showed efficacy in a subset of heavily pre-treated patients from diffuse large B-cell lymphoma (DLBCL) [88]. Different immune or cancer cells express CD39 which supports the tumor in escaping and immune recognition. Ectonucleotidases CD39 act in concert to convert extracellular immune-stimulating ATP to immunosuppressive adenosine. An interesting study has shown that treating tumor-bearing mouse models with CD39-specific ASO resulted in suppression of CD39 expression in a specific immune population such as Tregs and TAMs that help in tumor growth reduction [89].

**Fig. 3:**
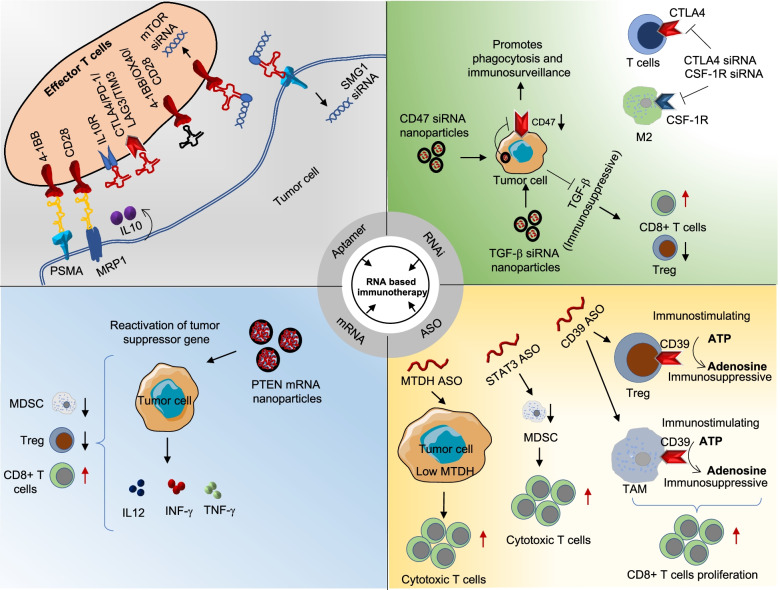
RNA-based modulation of the TIME and immunotherapy. Effector T cells, which play an important role in the antitumor immune response, can be targeted by different kinds of in vitro-transcribed RNA aptamers such as bispecific, antagonist, and chimeric. The interaction of these aptamers helps to prolong survival and inhibits T cell exhaustion that is linked with upregulation of antitumor immunity. Blocking immunosuppression-associated molecules using ASOs is another approach for cancer immunotherapy. Targeting immunosuppressor and immune-evading molecules such as CD47, TGF-β, CTLA-4, and CSF-1R by delivering their specific siRNAs in the form of NPs could lead to strong antitumor immune response. CD39 ASO downregulates CD39 expression on Tregs and TAMs, which are responsible for conversion of immunostimulating ATP into the immunosuppressive molecule adenosine. Modulating the function of MDSCs using STAT3 ASO results in the recruitment of higher levels of cytotoxic T cells. Targeting MTDH in the tumor cells with ASOs promotes antitumor immune response. Activating the tumor suppressor genes such as PTEN using PTEN mRNA-loaded NPs helps to inhibit the tumor growth by downregulating MDSCs and Tregs and activating CD8 + T cells. TGF-β, transforming growth factor-β; TNF-α, tumor necrosis factor α; INF-γ, Interferon gamma; IL, interleukin; IL-10R, interleukin-10 receptor; PSMA, prostate-specific membrane; LAG3, lymphocyte activation gene 3 protein; PD-1, programmed cell death protein 1; TIM3, T cell immunoglobulin mucin receptor 3; CSF-1R, colony-stimulating factor 1 receptor; ATP, adenosine triphosphate

Many ASOs have shown their promising outcomes under in vitro conditions and have been tested in clinical trials. However, no such ASO has received approval as a therapy in cancer yet. There are three ASO that have been granted orphan drug designation: oblimersen for chronic lymphocytic leukemia, cobomarsen for cutaneous T-Cell lymphoma and PNT2258 for DLBCL [90]. Overall, these studies indicated that ASOs are promising RNA-based therapeutic regimens for improving RNA-based immunotherapy (Fig. 3).

### RNAi and TIME

RNAi is an innovative gene silencing approach that is based on the delivery of double-stranded non-coding RNA (dsRNA) into cancer cells. This dsRNA triggers RNA-induced silencing complex-dependent oncogenic RNA degradation. The diverse approaches to induce RNAi include siRNAs, short hairpin RNAs (shRNAs), miRNAs, piwi-interacting RNAs, and lncRNAs. RNAi techniques have been shown to effectively target specific genes in different kinds of cells from the TIME.

#### siRNA

There are several methods of delivering siRNA or shRNA into cells depending on the model system. The introduction of siRNA is widely divided into three groups: naked siRNA delivery, lipid formulation-based delivery, and conjugate delivery. Generally, these molecules are delivered through the bloodstream or locally introduced into tumor tissue. However, there are many setbacks, including short half-life, rapid clearance from the blood circulation by the phagocytic system, instability, toxicity, off-target effects, and cellular permeability. To avoid such setbacks, and to increase the efficiency and safety of the treatment, siRNA-based NPs and lipid conjugate-based RNAi delivery can be used [91]. Recently, Kampel et al. determined the therapeutic potential of anti-E6/E7 HPV oncoprotein siRNA in human papillomavirus (HPV)-induced cancer in a xenograft tumor model. In their study, lipid-based NPs were used as siRNA delivery vehicles at the target site both in vitro and in vivo. This approach demonstrated high suppression of HPV oncogenes and induction of apoptosis activity, resulting in antitumor activity [92]. This finding provided a foundation for targeting various key regulators involved in the TIME (Fig. 3).

Recently, tumor cell-targeted siRNA-mediated immunotherapy revolves around targeting the TIME. To stimulate antitumor immune responses by downregulating ICP proteins, anti-inflammatory cytokines, and key immune signaling molecules are being targeted. For example, CD47 is an ICP protein overexpressed on the tumor cell surface that provides a “don’t eat me” signal to phagocytic cells such as macrophages. It inhibits SIRP1α and enables tumor cells to escape from immunosurveillance. A siRNA targeting CD47 on tumor cells was systematically delivered by HA-coated lipid NPs into melanoma cancer cells, resulting in CD47 knockdown, which facilitated phagocytosis and led to the inhibition of melanoma growth and metastasis [93]. Similarly, Xu et al. delivered a siRNA against immune-suppressive cytokine TGF-β encapsulated in mannose-modified liposome-protamine-hyaluronic acid NPs (LPH) into B16F10 melanoma tumor cells [94]. To improve the efficacy, they have delivered tumor antigens (i.e., Trp 2 peptide and CpG oligonucleotide) by lipid-calcium-phosphate NP (LCP) into the dendritic cells that trigger the systemic immune response. The in vivo experiment suggested that knockdown of TGF-β by LPH enhanced the vaccination efficacy of LCP as a result of decreased Treg levels and increased levels of tumor-infiltrating CD8 + T cells that significantly suppressed tumor growth [94]. The enhanced antitumor effect conferred by combining two NPs indicated that two or more therapeutics can influence antitumor immune response and offer a better platform for cancer immunotherapy. On the basis of this finding, RNA-based therapeutics with photodynamic or chemical mediators are being explored [31, 95]. Wang et al. have shown that, when delivered to tumor cells, a pH-responsive nanosystem co-loaded with PD-L1 siRNA along with a mitochondrion-targeting photosensitizer showed synergy in inhibiting tumor growth and metastasis in B16-F10 melanoma model [31]. A comparable study has revealed that a ROS-responsive nanotheranostic in combination with temozolomide chemotherapy and TGF-β siRNA-based immunotherapy exerted antitumor immune response in glioblastoma [96]. Both in vitro and in vivo data confirmed that these nanotheranostic NPs successfully reduced TGF-β expression in tumor cells and markedly boosted the efficacy of temozolomide-mediated chemotherapy [96]. Another approach, instead of silencing immunosuppressive genes, knockout, or knockdown of oncogenic genes via siRNA and ICB therapy could offer an effective means of cancer treatment. For instance, extracellular vehicles have been developed as a biological NP-mediated delivery system for the intrahepatic transfer of β-catenin siRNAs into hepatocellular carcinoma [97]. Under in vitro and in vivo conditions, systemic administration of extracellular vehicles containing β-catenin siRNA in combination with anti-PD-1 therapy improved CD8 + T cell infiltration and priming, increasing the antitumor effect of anti-PD-1 therapy [97].

The NP-siRNA-based delivery system could alter the immune cell milieu in the TIME to improve antitumor response. For example, targeting CTLA-4 using the NP-CTLA-4-siRNA system increased the percentage of tumor-infiltrating CD8 + T cells and decreased the level of Tregs, resulting in amplified activation and antitumor immune responses [98]. Another study showed that depletion of colony-stimulating factor-1 receptor (CSF-1R) using siRNA in M2-like TAMs caused their depletion in the melanoma TIME [99]. Importantly, these advanced siRNA delivery systems could inhibit immunosuppressive factors, including IL-10 and TGF-β, and in parallel upregulate immunostimulatory cytokines, such as IFN-γ and IL-12, along with CD8 + T cell infiltration in the TIME. Additionally, siRNA therapy successfully stimulated the antitumor function of T cells by downregulating the exhaustion markers PD-1 and T cell immunoglobulin mucin receptor 3 (TIM3) and stimulating the secretion of IFN-γ, dramatically inhibiting tumor growth and prolonging survival [99]. DCs expresses inhibitory molecules that suppress their antigen presentation activity, including suppressors of cytokine signaling 1 (SOCS1), STAT3, and indoleamine 2,3-dioxygenase. Studies have shown that downregulation of these inhibitory components by RNAi tools is highly effective for targeting DCs-based immunotherapy [100–104]. To this end, SOCS1 siRNA combined with tumor antigens ovalbumin encapsulated in poly lactide-co-glycolic acid polymeric NPs were delivered to DCs and showed enhanced antitumor immune response [105]. Several reports have suggested that MDSCs are essential to immunosuppression; hence therapeutic strategies to eliminate these cells or to modulate their functions are being explored [106–109]. In study led by Leuschner et al. delivered CCR2 siRNA using inflammatory monocyte-targeting lipid NPs [110]. The results indicated significant inhibition of CCR2 expression in monocytes, restricted their accumulation in inflammatory sites, and reduced the number of TAMs. siRNA technology has been effectively employed in mouse models to elucidate the role of master regulator genes of immune responses. As a proof of concept, this approach can turn intracellular checkpoints into therapeutic targets. For example, APN401 is siRNA specifically targeting the E3 ubiquitin ligase CBLB, an intracellular molecular adaptor that suppresses T-cell activation and boosts the anticancer immune response [111, 112]. Even with potential immunomodulatory effects of siRNA-based treatment approach, the only RNAi drug, rintatolimod, showed promising results in phase 1 and 1/2 clinical trials and can be used for the treatment of HER2 + breast cancer, triple-negative breast cancer, and several other solid tumors such as RCC, pancreatic cancer, and ovarian cancer. Rintatolimod is a toll-like receptor 3 (TLR3) agonist and activates interferon-induced proteins that require dsRNA for their activity. In addition, some reports suggest that TLR3 agonists reactivated the local innate immune response in NSCLC patients [113, 114].

Despite great success, siRNA-based approaches have been ineffective in clinical settings due to less stability and difficult delivery methods. Unprotected siRNAs have low stability in serum, and they are easily disrupted by endonucleases. In addition, kidneys can rapidly filter out siRNAs that are not fused with NPs. Hence, improvements are needed to reduce the barriers associated with the siRNA-based approach for anticancer immunotherapy.

### miRNAs and TIME

miRNAs are ~ 18–22 nucleotide short non-coding RNAs that regulate the stability and degradation of mRNA using natural RNAi machinery. The mechanism by which miRNAs and siRNAs regulate the expression of target transcripts is analogous and uses an RNA-induced silencing complex on the target transcript. siRNAs precisely degrade or inhibit mRNA translation with 100% complementarity in contrast to miRNAs, which can interact with incomplete complementarity sequences to perform gene silencing through slicer-independent pathways [115].

miRNAs exhibit a significant molecular mechanism for the crosstalk between tumor and immune cells by influencing immune cell functions in the TIME [116]. Importantly, functions of miRNA in cancer immune surveillance and escape have also been demonstrated [117]. Numerous studies have implicated tumor-suppressor miRNAs in regulating antitumor immune response within the TME by controlling ICPs such as PD-1, PD-L1, and CTLA-4 [118–121]. PD-1 or PD-L1 are individually targeted by some of these miRNAs, but both PD-1 and PD-L1 are simultaneously targeted by others, including miR-33 and miR-BART cluster [122, 123]. miRNAs can regulate functions of key immune cells, including macrophages, MDSCs, and NK cells, and contribute to tumor antigen processing for MHC-restricted presentation. It is believed that miRNAs may have a prognostic role in the setting of anticancer immunotherapy [122].

#### miRNA mimetics

Two main strategies have been employed in miRNA-based therapeutics: first, the development of miRNA analogues for miRNAs that have cancer-inhibiting effects, and second, the use of ASOs, LNAs, or antagomiRs to block miRNAs that have oncogenic effects. miRNA mimetics are RNA-based small molecule drugs that expand the therapeutic hits for cancer immunotherapy. This approach was efficiently confirmed in vivo by the delivery of an miR‑155 mimetic that changed the phenotype of tumor associated DCs to a pro-inflammatory phenotype that stimulated antitumor immune responses [124]. In another study, longitudinal blood samples from mice and patients with lung cancer treated with PD-1 inhibitors showed enrichment of exosome miRNA-4315, which induces apoptosis resistance to chemotherapy by downregulating expression of the pro-apoptotic protein Bim. The incorporation of ABT263 (a BH3 mimetic) evaded this resistance [125]. This study demonstrated an alternative therapeutic opportunity to use miRNAs for patients with anti-PD1-resistant and provides a chance to modulate immunotherapy. Cancer immunotherapy mediated by miRNAs also may benefit patients with neuroblastoma. Neviani et al. showed that exosomes derived from NK cells carrying cancer suppressor miR-186 in an in vivo orthotopic model of neuroblastoma prohibited growth, proliferation, and TGFβ-dependent immune escape mechanisms [126].

### LncRNAs as TIME modulator

LncRNAs are ubiquitously expressed non-protein-coding transcript, > 200 bp in length. LncRNAs are localized in the cell nucleus, cytoplasm, and exosomes and form a complex regulatory network with various molecules such as DNA, RNA, and proteins [127]. They are reported to be involved in pathophysiological processes through the epigenetic, transcriptional, and post-transcriptional regulation of gene expression [128]. LncRNA regulates immune response via multiple pathways (e.g., NF-κB/MAPK, JAK/STAT) that control the differentiation, development, and effector functions of immune cells [129, 130]. For example, lnc-DC, a lncRNA expressed in DCs, was found to be required for DC maturation and secretion of cytokines, including IL-6, IL-12, and IFN-γ [131]. Another example includes GAS5, which regulates the killing effect of NK cells and requires NK-dependent antitumor function [132]. At the TME level, lncRNAs are involved in controlling interaction between immune cells and tumor cells and induce the immunosuppressive microenvironment [133]. For instance, the upregulation of lnc-TIM3 in tumor-infiltrating CD8 + T cells prevents IFN-γ and IL-2 production and leads to T cell exhaustion in the TME [134]. Tumor immunity-associated lncRNAs are mainly localized in specific types of cancer cells or stromal cells. Most studies of lncRNA in the TME are focused on T cells and MDSCs that determine lncRNAs’ role in disease progression and immune response regulation [135, 136]. Additionally, studies have shown that lnc-DC plays an important role in DC differentiation and stimulates T cell activation during tumor immune response [137]. These reports are restricted to preclinical and clinical applications of lncRNA-mediated cancer immunotherapy [133]. LncRNAs that regulate the pivotal molecules and pathways during cancer progression are targeted in anticancer therapies, such as therapeutic vaccines, T cell-based treatments, and ICB. Several lncRNAs also might predict tumor immunotherapy response [135, 136]. The efficacy of ICB therapy depends on T cell-recognized neoantigens displayed by MHCs on tumor cells. Thus, the absence of tumor neoantigen recognition leads to resistance to PD-L1/PD-1 inhibitors. Several well-studied immune-related lncRNAs have been found to act as key factors during tumor immune response at the epigenetic level. Importantly, lncRNAs can act both as an activator or repressor of immune response genes (Fig. 3). Thus, lncRNAs involved in the regulation of antigen presentation or ICB may serve as therapeutic targets.

Several reports have shown that lncRNAs Neat1 and Malat1 exhibit a potential role during tumor progression and metastasis [138–140]. Targeting these lncRNAs using their specific ASOs significantly reduced tumor growth and metastasis. However, these lncRNAs’ correlation with TIME modulation is largely unexplored [138, 140]. In a preclinical study, it has been reported that attenuation of Neat1 can inhibit apoptosis and enhance antitumor cytolytic activity in CD8 + T cells [141]. Moreover, TIMER database analysis from Guo et al. has indicated a major role of Malat1 in T cells development and function in patients with various cancer types [142]. Mouse models of breast cancer have shown high enrichment of LINK-A lncRNA expression in mammary gland tumors, and depletion of LINK-A expression repressed tumor progression [143]. Another mouse model found that combination treatment with LINK-A LNA-based ASOs and ICB synergistically reduced tumor growth and increased survival [143]. Therefore, LINK-A shows a promising biomarker for outcomes in patients who have triple-negative breast cancer and are treated with ICB. LncRNA in combination with CAR T cell-based therapy is used as an adjuvant for the epigenetic regulation of T cell apoptosis. For example, NKILA has been shown to sensitize antitumor T cells to cell death upon activation by tumor antigens [144]. In immunocompromised mice, CD8 + CTLs transduced with NKILA shRNA were administered along with human breast cancer xenografts and this effectively inhibited tumor growth. CTL cytotoxicity and anti-apoptotic gene expression levels were higher in tumors from shRNA-treated mice than in those from control mice [144]. NKILA inhibition in tumor-infiltrating lymphocytes and CAR T cells may silence their activation-induced cell death, thus suppressing tumor immune evasion and expanding the efficacy of cancer immunotherapy [144].

### CircRNAs and TIME

CircRNAs are another class of non-coding RNAs that are single-stranded and covalently closed at the 3’ and 5’ ends, forming a hoop-like structure. Due to their covalently closed structure, circRNAs are resistant to RNAse treatment and hence highly abundant and stable in the cytoplasm. Several genes produce circRNAs by an alternative RNA splicing mechanism called back-splicing [145]. Recent advancements in sequencing along with extensive studies and several circRNA databases helped us to gain detailed insight into the functionality of circRNAs. For instance, Vo et al. have done exome capture transcriptome sequencing and established a cancer circRNA landscape. Based on these studies, they created MiOncoCirc, the first database primarily composed of circRNAs directly detected in tumor tissues [146]. CircRNAs are predicted to be a biomarker for certain cancer types, but their full potential as a therapeutic target in the TME has not been realized. Like lncRNAs, circRNAs can be employed in anticancer immunotherapy as tumor antigens or vaccine adjuvants. CircRNAs can also be used to suppress onco-miRNAs by sponging/sequestrating them into the targeted cancer cells, thus inhibiting a crucial regulator of carcinogenesis [147]. An interesting study suggests that SLC8A1 gene-derived circSLC8A1 helps in the migration of MDSCs to the tumor site and enhances the tumor immune response. It regulates the production of ARG1 and iNOS by acting as a sponge of miR-494, which is essential for MDSCs’ migration [148]. Additionally, it has been shown that circARSP91 could enhance the NK cells’ cytotoxicity by upregulating UL16-binding protein in hepatocellular carcinoma cells [149]. Moreover, hsa_circ_0020397 and circ_0000284 have related to PD-L1 expression [150]. Moreover, circRNA-100783 is involved in CD8 + T cell aging and immunosenescence, while circRNA-003780 and circRNA-010056 have functions in macrophage differentiation and polarization [151]. Interestingly, it has been discovered that circRNA is a potent immuno-stimulant that could be used as an adjuvant in a vaccine setting [152]. CircRNAs can also be delivered to cells by advanced techniques, such as exosomes or viroids. The incorporation of circRNAs into target cancer cells using recently developed techniques can affect the communication/signaling between stromal and tumor cells during tumorigenesis, and some promising data are already validated [153]. Despite of all these novel and advanced research on circRNAs, still these are in its infancy to be incorporated in clinical practices, as the circRNAs mediated regulatory networks are very complex. CircRNA could be tumor- and TME type-dependent, which would provide new avenues for RNA-based immunotherapy (Fig. 3).

### RNA aptamers and immunotherapy

Aptamers are single-stranded oligonucleotide ligands folded in complex three-dimensional assemblies. They can bind their targets with high affinity and specificity by non-covalent pocket interactions and block essential interactions between the target and other molecules. Unlike antibodies, these are small molecules with different pharmacokinetic properties. Aptamers are short-lived, having a half-life of 24–48 h compared to antibodies, which have a half-life of 2–4 weeks hence, aptamers were first used in localized applications [154]. Currently two aptamers, NOX-A12 and NOX-E36, targeting immunosuppressive chemokines in the TME are being tested in clinical studies of anticancer immunotherapy. NOX-A12 was developed against stromal cell-derived factor 1 (SDF1) is being used in combination with pembrolizumab in pancreatic and colorectal cancer patients [33]. NOX-A12 administration increased T and NK cells infiltration in preclinical models of colorectal cancer [155]. Moreover, combined treatment with NOX-A12 and PD1 blockade showed synergistic behavior in T cell activation. Another aptamer, NOX-E36 targets C–C chemokine ligand 2 (CCL2) to suppress migration and infiltration of immunosuppressive macrophages and MDSCs in solid malignancies. Based on their function, aptamers can be divided into three main categories, which are described below.

#### Antagonistic aptamers

Antagonistic aptamers inhibit the interaction between a receptor and its ligand. Most therapeutic aptamers fall into this category. Additionally, the use of antagonistic aptamers to modulate the TIME is growing. The first therapeutic aptamer was designed against CTLA-4. Upon tetramerization this aptamer inhibited CTLA-4 functions profoundly and displayed in vivo antitumor effects through enhancing antitumor immunity [156]. It is well established that IL-10 is an immunosuppressive cytokine secreted in the setting of high tumor burden, but its function can be hampered by interrupting its binding with the IL-10 receptor (IL-10R). To this end, an IL-10R antagonist aptamer was developed, which can activate T cells and mediate antitumor immune responses [157]. It has been shown that lymphocyte activation gene 3 protein (LAG3) and TIM3 receptors are expressed with PD-1 in exhausted T cells. Also, the blockade of these ICPs was sensitized by suppressing non-overlapping immunosuppressive pathways in T cells. Hence, it makes sense to select TIM3 and LAG3 antagonist RNA aptamers by RNA-based libraries via systematic evolution of ligands by exponential enrichment (SELEX) technology [158, 159]. In a mouse model, to decrease immunosuppressive MDSCs infiltration in tumors, an aptamer against C5a anaphylatoxin chemotactic receptor (C5aR) was combined with PD-1 blockade, showing synergy in inhibiting KRAS-driven lung cancer [160].

#### Bispecific aptamers

To achieve maximum therapeutic value, aptamers can be multimerized. These multimers can have dual specificity to recognize different target molecules concurrently. An interesting example is the bispecific aptamer designed against 4-1BB, which was conjugated with an anti-human prostate-specific membrane antigen (PSMA) aptamer. PSMA is a transmembrane protein is expressed in all prostate cancers, and its expression correlates with metastasis. 4-1BB co-stimulation is required for the infiltration of active T cells in the tumor, and administration of 4-1BB agonistic antibodies alone can elicit liver inflammation [161]. As a proof of concept, genetically modified tumor cells that externally expressed a membrane non-internalizing PSMA-targeting receptor were used to administer this bispecific aptamer. In two different tumor models expressing PSMA, PSMA-4-1BB aptamers showed a higher therapeutic index compared with a monoclonal 4-1BB agonistic antibody [162, 163]. A similar approach was adopted in the TME. An aptamer targeting vascular endothelial growth factor was linked to a 4-1BB aptamer to activate infiltration of T cells by co-stimulation [164]. Likewise, a bispecific aptamer (MRP1-CD28) was used to guide CD28 agonistic aptamers to cancer stem cells expressing multidrug resistance-associated protein 1 (MRP1, also called ABCC1), which is ubiquitously expressed in aggressive tumors [165]. Bispecific aptamers are a better option to increase the therapeutic index of immunostimulatory agents.

#### Aptamers as target ligands

The combinatorial approach conjugating aptamers with siRNAs to block signal pathways within a specific cell type is being investigated. The first aptamer-siRNA conjugate was a PSMA aptamer conjugated with polo-like kinase 1 (PLK1) siRNA in PSMA-expressing prostate tumors [166, 167]. In line with this, 4-1BB aptamers were exploited to transfer siRNA into T cells to interrupt the mTOR pathway and IL-2 receptor (IL-2R) signaling, a deciding factor for determining the fate of memory T cells [168, 169]. Furthermore, a CTLA-4 aptamer employed with immunosuppressive transcription factor STAT3 siRNA indicated a drop in Tregs infiltration and displayed antitumor effects in mice [170]. In another approach, instead of using siRNA, a forkhead box protein P3 (FOXP3)-blocking peptide was attached to a CD28-targeting aptamer. This resulted in the inactivation of Treg function and improved outcomes of RNA aptamer-based cancer immunotherapy (Fig. 3) [171].

Aptamer therapy has some clinical benefits over antibodies that are worth mentioning. First, it gives the opportunity to manage adverse side effects or reduce immunological risks in clinical trials [172, 173]. Second, clinical-grade manufacturing or production of these aptamers is much simpler and cost-effective. Third and most importantly, these aptamers can be chemically attached with different therapeutic entities and delivered to specific sites/cells with ease [174]. Finally, aptamers are almost lacking in antigenicity and hence are unlikely to encourage neutralizing T cell-dependent humoral immune responses, which are usually initiated by protein-based therapeutic agents.

## Current challenges and future perspectives

Various RNAi mechanisms that inhibit and target immunosuppressive activities in the TME are slowly gaining attention. However, more efficient delivery approaches for in vivo applications are needed, especially for systemically delivering RNA therapeutics into immune cells associated with the TIME. There are still some challenges in the field of targeting the TIME via RNA-based therapeutics. First, the spatial architecture of the TIME for multiple tumor types remains unresolved. The spatial proximity between tumor and immune cells does not necessarily mean that actual communication is happening. Second, RNA-based therapeutics lack efficacy, specificity, and selectivity. Several technological advancements are likely to happen in this field; among these are chemically modified RNA molecules with specific structures instead of in vitro-transcribed RNA. Due to nano chemistry, in vivo RNA delivery methods into various immune cells are being developed. Currently, cancer immunotherapies are heavily dominated by ICB techniques, antibody/oncoprotein technologies, CAR T cells, and small molecules yet these approaches are accompanied by several disadvantages. Hence there is still scope for improvement with RNA-based immunotherapy combined with other traditional methods. In recent years, with increased knowledge about miRNAs, more and more combination therapies are being explored. Promising clinical trials with RNA-based therapies and vaccines will gain interest in the near future and will provide an opportunity with pharmacokinetic, cost, and regulatory advantages.

It is worth highlighting that most RNA therapeutics have promising applications in cancer research concerning tumor immunotherapy. However, the studies covered in the review provide us a basis for exploring new biomarkers or candidates for RNA therapeutics that can expand the clinical applications of immunotherapy. Additionally, advanced studies are required to explore RNA-based approaches for treating cancer, which are far from perfect. For instance, new computational methods are required to discover cancer-associated lncRNAs or circRNAs. Moreover, lncRNA- or circRNA-specific animal models should also be established to enable a better understanding of the roles of lncRNAs/circRNAs within the TIME for clinical applications.

It is expected that in near future, both pros and cons will be attached to RNA-based immunotherapy. More researchers from RNA technology, along with clinicians and cancer biologists, will have to devote themselves to advance RNA antitumor therapy. In this cutting-edge research of cancer immunotherapy, future combinations of RNA-based immunotherapies will be a matter of investigation.

## Conclusion

In this review article, we have demonstrated diverse RNA-based therapeutics that have direct or indirect implications in the modulation of the immunosuppressive TIME. Based on growing discoveries in RNA-based cancer therapeutics, the new strategies will have an impact on the treatment of cancer and other diseases that previously had limited or no treatment options. After decades of siRNA therapy development, targeting and delivery of these therapeutics have improved significantly. Although various RNAi drugs have been developed, only a handful have completed phase 1 clinical trials. Obstacles such as stability, stimulation of innate immune stimuli, off-target effects, and safety concerns continue to limit siRNA-based drugs, and studies will be needed to address these obstacles. RNAi therapy, which exerts its effect through gene silencing, will likely enable faster and better treatment of diseases such as cancer.

## Data Availability

Not applicable.
